# Negative Modulation of TRPM8 Channel Function by Protein Kinase C in Trigeminal Cold Thermoreceptor Neurons

**DOI:** 10.3390/ijms21124420

**Published:** 2020-06-22

**Authors:** Bastián Rivera, Matías Campos, Patricio Orio, Rodolfo Madrid, María Pertusa

**Affiliations:** 1 Department of Biology, Facultad de Química y Biología, Universidad de Santiago de Chile, and Millennium Nucleus of Ion Channel-Associated Diseases (MiNICAD), 9160000 Santiago, Chile; bastian.rivera@usach.cl (B.R.); matias.campos@usach.cl (M.C.); rodolfo.madrid@usach.cl (R.M.); 2 Centro Interdisciplinario de Neurociencia de Valparaíso and Instituto de Neurociencia, Facultad de Ciencias, Universidad de Valparaíso, 2360102 Valparaíso, Chile; patricio.orio@uv.cl

**Keywords:** primary sensory neurons, corneal nerve endings, cold transduction, plasma membrane, PMA, menthol

## Abstract

TRPM8 is the main molecular entity responsible for cold sensing. This polymodal ion channel is activated by cold, cooling compounds such as menthol, voltage, and rises in osmolality. In corneal cold thermoreceptor neurons (CTNs), TRPM8 expression determines not only their sensitivity to cold, but also their role as neural detectors of ocular surface wetness. Several reports suggest that Protein Kinase C (PKC) activation impacts on TRPM8 function; however, the molecular bases of this functional modulation are still poorly understood. We explored PKC-dependent regulation of TRPM8 using Phorbol 12-Myristate 13-Acetate to activate this kinase. Consistently, recombinant TRPM8 channels, cultured trigeminal neurons, and free nerve endings of corneal CTNs revealed a robust reduction of TRPM8-dependent responses under PKC activation. In corneal CTNs, PKC activation decreased ongoing activity, a key parameter in the role of TRPM8-expressing neurons as humidity detectors, and also the maximal cold-evoked response, which were validated by mathematical modeling. Biophysical analysis indicated that PKC-dependent downregulation of TRPM8 is mainly due to a decreased maximal conductance value, and complementary noise analysis revealed a reduced number of functional channels at the cell surface, providing important clues to understanding the molecular mechanisms of how PKC activity modulates TRPM8 channels in CTNs.

## 1. Introduction

Cold transduction occurs at the free nerve endings of cold thermoreceptor neurons (CTNs), a subpopulation of primary somatosensory neurons of the dorsal root ganglia (DRG) and trigeminal ganglia (TG) that innervate the skin and exposed mucosae (reviewed by [1,2,3,4]). Trigeminal CTNs are not only involved in cold sensing; they are also part of the neural circuit that regulates ocular surface wetness by adjusting both the basal tearing rate and spontaneous blinking frequency [5,6,7,8,9]. These two physiological functions rely on the exquisitely thermosensitive machinery at their peripheral nerve terminals, where the main molecular transducer for innocuous cold temperatures is the Transient Receptor Potential Melastatin 8 (TRPM8) ion channel [5,6,10,11,12]. TRPM8 is a Ca^2+^-permeable non-selective cation channel activated by cold, cooling compounds such as menthol, voltage, and discrete osmolality increases [6,13,14,15,16]. Functional dysregulation of TRPM8 has been linked to diverse physiopathological states, including neuropathic and inflammatory pain, and several forms of cancer (reviewed by [17,18,19,20,21,22,23]). Thus, understanding the molecular bases underlying altered TRPM8 function is an attractive first step in the search for future clinical strategies. TRPM8 is diversely regulated in both physiological and pathological conditions by interacting proteins [24,25], splice variants [26], post-translational modifications [27,28,29,30,31], modulatory regions [32] and G protein-coupled receptor signaling cascades [33,34,35,36,37,38]. The latter is particularly relevant during tissue injury and inflammation, where released inflammatory mediators enhance thermal-evoked pain sensation. Although thermal sensitization during inflammation is mainly related to the upregulation of the heat-gated TRPV1 channel [39,40], some authors have also described the effect of inflammatory agents on CTNs responses [33,34,35,37,38]. Different studies agree that DRG and TG neurons express G protein-coupled receptors for proinflammatory mediators that significantly downregulate TRPM8 function, such as bradykinin and prostaglandin E2 [33,34,35,37,38]. Most of these receptors, including bradykinin receptor 2 (BR2), are G_αq_-coupled receptors that lead to the subsequent activation of phospholipase C (PLC) and PKC.

Several studies that explore the different molecular components of this signaling cascade observe a complex scenario, where three different mechanisms could explain the negative modulation of TRPM8: depletion of phosphatidylinositol 4,5-bisphosphate (PIP_2_) [33,41,42], that acts as an obligatory cofactor in TRPM8 activation [41,42,43,44]; direct interaction of activated G_αq_ protein with TRPM8 [34,38]; and activation of PKC [35,37]. Among them, PKC-dependent modulation of TRPM8 is still controversial. Whereas some studies conclude that PKC activation by proinflammatory mediators modulates TRPM8 [35,37], other authors argue against a meaningful contribution of this kinase in the functional downregulation of this channel [33,34,38]. Moreover, reports that focused on the effect of PKC activators on TRPM8 function, such as phorbol esters, also reveal contradictory observations. Although most studies that explore PKC-dependent modulation show a robust reduction in the responses of TRPM8 channels to agonists after treatment with phorbol 12-myristate 13-acetate (PMA) and phorbol 12,13-dibutyrate (PDBu) [35,37,45,46], some authors did not observe this effect [34].

TRPM8 has a key role in excitability disturbances of corneal fibers induced by peripheral nerve damage. In corneal physiology, local inflammation caused by surgical procedures (photorefractive or cataract surgery), tear film pathologies (dry eye disease) or long-term wearing contact lenses, among others, affect the peripheral axons and nerve terminals of corneal sensory neurons, finally altering ocular surface homeostasis [9,47,48,49,50]. Since tissue damage and inflammation can trigger signaling pathways that lead to PKC activation (reviewed by [51]), PKC-dependent regulation of TRPM8 function in corneal nerve endings is an exciting opportunity to explore its role in a physiologically relevant context.

Considering that there is no consensus regarding the role of PKC in TRPM8 function, in this study, we have revisited this question. We assessed the effect of the PKC activator PMA on TRPM8-dependent responses in a heterologous expression system and in native membranes, including TRPM8-expressing corneal cold thermoreceptors ex vivo, where even subtle alterations of the TRPM8 activity can induce a substantial physiological impact. This study also aims to gain further insight into the molecular mechanism behind this form of regulation, exploring whether PKC activation modifies the density of active channels in the plasma membrane, alters TRPM8 biophysical properties, or both.

Our results indicate that PKC acts as a negative modulator of TRPM8, mainly by decreasing the presence of functional channels at the cell surface, and that PKC-activation in corneal CTNs reduces their spontaneous firing and maximal response to temperature drops, suggesting that this kinase is not only relevant to the modulation of cold sensing but also in ocular wetness regulation.

## 2. Results

### 2.1. PKC Activation Reduces the Responses of TRPM8 Channels Expressed in HEK-293 Cells to Cold and Menthol

**Figure 1:**
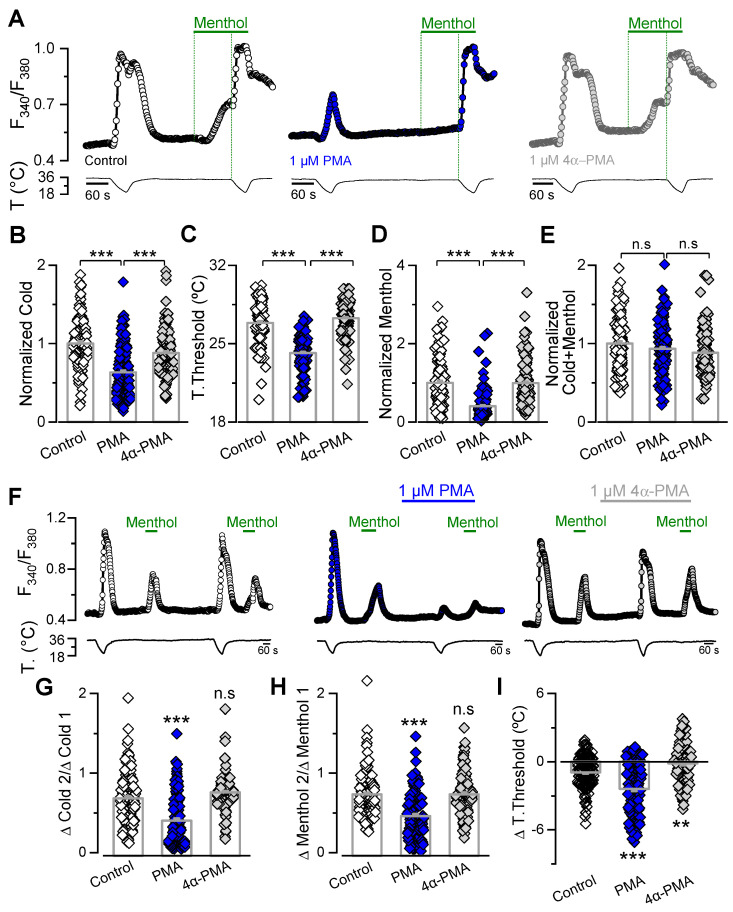
TRPM8 responses to cold and menthol are reduced by PMA-induced PKC activation. (**A**) Representative ratiometric [Ca^2+^]_i_ traces showing responses of HEK-293-mTRPM8-myc stable cell line to cold, 100 µM menthol, and cold in the presence of 100 μM menthol. Cells were preincubated for 10 min at 37 °C with control solution (left, open circles), 1 µM PMA (central panel, blue circles) and 1 µM 4α-PMA (right panel, grey circles). In each condition, the upper trace shows [Ca^2+^]_i_ and the lower trace represents bath temperature. Green vertical dotted lines indicate the start of menthol and cold plus menthol stimuli. (**B**–**E**) Bar graph representing mean and S.E. and data points of cold- (**B**), temperature threshold (**C**), menthol- (**D**), and cold+menthol-induced responses (**E**) for each condition. The values were normalized to the mean response observed in control condition. Control, *n* = 119; PMA, *n* = 149 and 4α-PMA, *n* = 146. Statistical significance was assessed using a one-way ANOVA test in combination with a Bonferroni’s post hoc test; *** *p* < 0.001 and n.s. (not significant) *p* > 0.05. (**F**) Representative Fura-2 ratiometric [Ca^2+^]_i_ traces showing responses of HEK-293-mTRPM8-myc cells to cold and menthol stimuli under control conditions (left panel) and before and following treatment with 1 μM PMA (central panel) or 1 μM 4α-PMA (right panel). (**G**,**H**) Bar graph representing mean and S.E. and data points of ratio responses to cold (ΔC2/ΔC1) (**G**) and menthol (ΔM2/ΔM1) (**H**) for each condition. (**I**) Summary of the effect of different treatments on the temperature threshold of cold-evoked responses (T2-T1). Negative values indicate shifts towards lower temperatures. Control, *n* = 134; PMA, *n* = 130 and 4α-PMA, *n* = 112. Statistical significance was assessed using a one-way ANOVA test in combination with a Dunnett’s post hoc test; *** *p* < 0.001, ** *p* < 0.01 and n.s. *p* > 0.05, compared to control.

To evaluate the ability of PKC to downregulate TRPM8 function, we used phorbol 12-myristate 13-acetate (PMA), a specific pharmacological activator of protein kinase C, in vivo and in vitro. This specific PKC activator was previously used in studies reporting evidence for and against the involvement of PKC in TRPM8 modulation [34,35,37,46]. As a first approach, we used Ca^2+^-imaging, a non-invasive technique that avoids a potential disruption of cell-signaling pathways. HEK-293 cells stably expressing mTRPM8-myc channels (HEK-293-mTRPM8-myc) [52] were preincubated for 10 min at 37 °C with extracellular solution containing 1 µM of PMA, or with control solution (Figure 1). To exclude the direct effects of PMA on TRPM8 channels, we also tested the effect of the biologically inactive analog 4α-PMA at the same concentration as the active compound. Our first goal was to evaluate if there was a PMA-mediated reduction in the TRPM8 responses to cold and menthol in the recombinant system. We used an experimental protocol that consists of a 34 to 20 °C cold pulse, followed by a 100 µM menthol application at 34 °C, and finally, to elicit the maximal response of the channel, a second cold pulse, in the presence of the chemical agonist, was applied (Figure 1A). Responses to cold and menthol were strongly affected by PKC activation. For cold stimuli, the ∆F_340_/F_380_ was 0.48 ± 0.01 (*n* = 119) in the control cells (i.e., cells preincubated with control solution) and 0.31 ± 0.01 after treatment with 1 µM PMA (*n* = 149; *p* < 0.001, unpaired *t* test). In the same cells, ∆F_340_/F_380_ responses to 100 µM menthol were 0.17 ± 0.01 in control condition, and 0.07 ± 0.01 (*p* < 0.001, unpaired *t* test) after treatment with the PKC activator. Figure 1B–E shows the results normalized to the values obtained from control cells, where the shift in the temperature threshold was observed only in cells treated with the active phorbol ester. In contrast to the marked PMA-mediated reduction in cold- and menthol-evoked intracellular Ca^2+^ elevations, the amplitude of TRPM8-dependent responses to the saturating stimuli (i.e., menthol plus cold) was similar in both conditions. Importantly, comparison of the mean responses elicited by cold or menthol in cells preincubated with the PKC activator, or its inactive analogue 4α-PMA, showed that PMA induced a marked reduction, suggesting that the downregulation of the channel is mainly mediated by a PKC-dependent mechanism (Figure 1B–E). In these experiments, once the preincubation time was over, treated cells were transferred to the recording chamber and perfused with control solution in the absence of 4α-PMA or PMA, challenging that the observed downregulation could be explained by a direct interaction between PMA and TRPM8.

We also investigated the responses of HEK-293-mTRPM8-myc cells using a double-pulse protocol, wherein we compared the basal response to cold and menthol before and after treatment with 1 µM PMA in the same cell. As observed in Figure 1F–H, cells perfused with either standard solution or 1 µM 4α-PMA exhibited a decrease in the amplitude of the second response to cold and menthol due to desensitization [42,53,54]. However, this reduction was larger in cells treated with PKC activator, resulting in a second response amplitude that is half that of the first response to the thermal or chemical stimulus (Figure 1G,H). Treatment with 1 µM PMA also produced a larger shift in the temperature activation threshold to lower temperatures (−2.4 ± 0.2 °C, *n* = 130) than that exhibited by cells treated with 4α-PMA (−0.1 ± 0.2 °C, *n* = 112; *p* < 0.001, *t* test) (Figure 1I).

Collectively, these results suggest that PKC activation reduces TRPM8 responses to cold and menthol, and agrees with previous studies that concluded that PKC activation using PMA is sufficient to downregulate TRPM8 activity in this recombinant system.

### 2.2. PMA Treatment Reduces the Number of Active TRPM8 Channels at the Plasma Membrane

**Figure 2:**
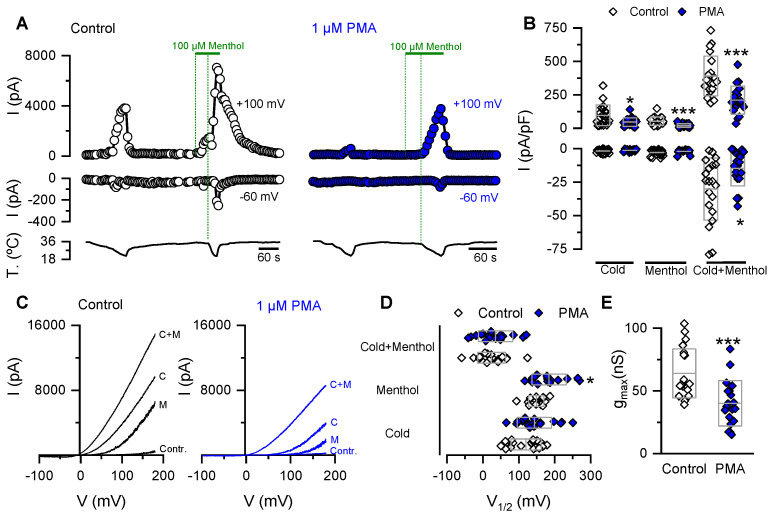
PKC activation reduces the *g*_max_ in HEK-293 cells expressing mTRPM8 channels. (**A**) Time course of whole cell current development at +100 and −60 mV in HEK-293-mTRPM8-myc stable cell line preincubated for 10 min with (right panel) or without (left panel) 1 µM PMA. (**B**) Scatter plot with mean ± S.D. of all values corresponding to the maximal current density at +100 and −60 mV. Mean whole-cell capacitance for control cells was 14.6 ± 0.6 pF, vs. 15.6 ± 0.6 pF for treated cells, *n* = 20 and 19, respectively; *p* > 0.05, unpaired Student’s *t* test. (**C**) Whole-cell *I–V* relationships at 34 °C in control solution (Contr.), at 20 °C in control solution (cold (C)), menthol at 34 °C (M), and menthol at 20 °C (C + M), for each condition. (**D**,**E**) Box plot with data points; the center line of the box represents the mean values, whereas the top and bottom of the box represent the SD of *V*_1/2_ values (**D**) and *g*_max_ estimated in the cold+menthol condition (**E**). Statistical significance was assessed with a two-tailed unpaired Student’s *t* test; * *p* < 0.05 and *** *p* < 0.001. Control, *n* = 20 and PMA, *n* = 19.

We performed patch-clamp experiments to gain further insight into the molecular bases of the PMA-induced reduction of cold- and menthol-evoked responses in cells expressing TRPM8. Under whole-cell configuration, HEK-293-mTRPM8-myc cells were held at −60 mV, and channel activation was tested using voltage ramps from −100 to +180 mV at 0.2 Hz to obtain the I-V relationships. We used the protocol in Figure 1A, consisting of sequential applications of a cold pulse, 100 µM menthol at 34 °C, and cold in the presence of menthol (Figure 2A). In agreement with our previous findings using Ca^2+^-imaging, preincubation of HEK-293-mTRPM8-myc cells for 10 min with PMA reduced the amplitude of the cold- and menthol-evoked currents (Figure 2A). A significant decrease in the maximum current density elicited by the cold plus menthol stimulus was also found using this more linear readout of channel activity (Figure 2B).

TRPM8 activation by its agonists is related to a shift of the voltage–activation curve towards more negative and physiologically relevant membrane potentials [15,16,53]. To estimate the midpoint of activation (V_1/2_) and the maximal conductance (g_max_) of the channel in our experimental conditions, the current traces derived from the voltage ramps (Figure 2C) were fitted with a Boltzmann-linear function (see Material and Methods, Equation (1)). Figure 2D,E shows that the PMA-dependent reduction in the TRPM8 response is due to a ~40% decrease in g_max_. A significant shift in the V_1/2_ of the voltage-dependent activation towards more positive membrane potentials was also observed, but only for menthol-dependent responses. These results suggest that the PKC-dependent drop of TRPM8 responses is linked to a large decrease in maximal conductance, especially for the most physiologically relevant (cold-dependent) form of TRPM8 activation.

**Figure 3:**
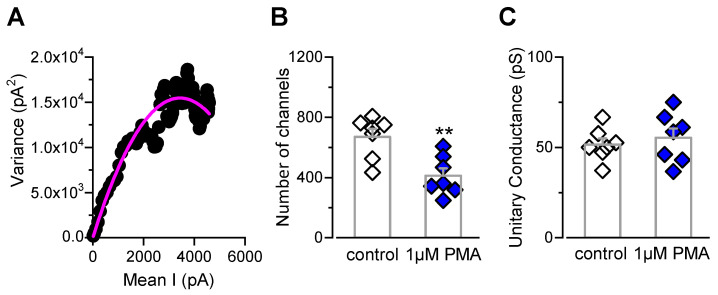
PKC activation by PMA induces a drop in the number of active TRPM8 channels at the plasma membrane. (**A**) Dot plot of variance versus mean current obtained from whole-cell currents of a HEK-293 cell stably expressing mTRPM8-myc channels, using 150 ms depolarizing voltage steps from 0 to +180 mV, at 19 °C. Solid line corresponds to fit of data to a parabola (Equation (2)). (**B**,**C**) Bar graph representing mean and S.E. and data points of the number of active channels (**B**) and unitary conductance (**C**) for cells preincubated for 10 min with 1 µM PMA (blue symbols) or control solution (white symbols). Statistical significance was assessed with a two-tailed unpaired Student’s *t* test; ** *p* < 0.01, *n* = 7 cells for each condition.

The reduction in the g_max_ value observed in patch-clamp experiments (Figure 2E) could be explained by a decreased TRPM8 channel density at the cell surface. To explore this hypothesis, we performed non-stationary noise analysis to estimate the number of functional TRPM8 channels at the plasma membrane (Figure 3). Figure 3A shows a representative dot plot of variance versus the mean current obtained from a control cell. We found that HEK-293-mTRPM8-myc cells in control conditions expressed 636 ± 61 channels, with a mean single-channel conductance of ~50 pS (*n* = 7 cells) (Figure 3B,C). These values are in line with other reports in similar conditions [29,55]. In contrast, incubation with the PKC-activator significantly decreased the number of channels to 391 ± 50 (*n* = 7 cells; *p* < 0.01, unpaired *t* test) (Figure 3B), without changes in the unitary conductance (Figure 3C). This result represents a ~40% reduction in the number of active TRPM8 channels at the plasma membrane due to PKC activation, consistent with the decrease in the g_max_ value reported in Figure 2E.

Altogether, these results suggest that PMA-dependent activation of PKC negatively modulates TRPM8 function, inducing a large drop in the number of functional channels at the cell surface.

### 2.3. PKC-Dependent Modulation of TRPM8 in CTNs from Trigeminal Ganglia

**Figure 4:**
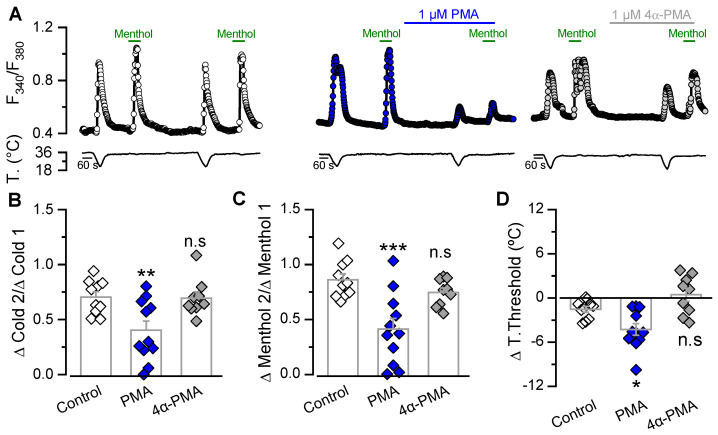
PKC activation by PMA downregulates the responses to cold and menthol in cold-sensitive neurons. (**A**) Ratiometric [Ca^2+^]_i_ response of representative cold thermoreceptor/menthol-sensitive neuron, from mouse TG. The protocol used consisted of a double application of a cold pulse followed by application of 100 µM menthol at 34 °C in cells perfused with control solution (open circles), 1 µM PMA (blue circles) or 1 µM 4α-PMA (grey circles). (**B**,**C**) Bar graph representing mean and S.E. and data points of ratio responses to cold (ΔC2/ΔC1) (**B**) and menthol (ΔM2/ΔM1) (**C**) for each condition. (**D**) Summary of the effect of different treatments on the temperature threshold of cold-evoked responses (T2-T1). Negative values indicate shifts towards lower temperatures (control, *n* = 10; PMA, *n* = 11 and 4α-PMA, *n* = 9). Statistical significance was assessed using a one-way ANOVA test in combination with a Dunnett’s post hoc test; *** *p* < 0.001, ** *p* < 0.01 and * *p* < 0.05, n.s. (not significant) *p* > 0.05 compared to control conditions.

Most reports that evaluate changes in response to cold and menthol induced by PMA in CTNs use cultured DRG neurons [34,35,37]. Although TG and DRG neurons are considered very similar, there are some important differences [56]. Since we wanted to explore the relevance of this modulation in the nerve endings of CTNs innervating the cornea, we first determined whether these alterations in cold and menthol-evoked responses are also seen in native channels expressed in dissociated TG neurons from adult mice. Figure 4A shows changes in the intracellular Ca^2+^ concentration in trigeminal CTNs exposed to a cold pulse and a 100 µM menthol application, before and after incubation with control solution, 1 µM PMA or 1 µM 4α-PMA. In tight correlation to our observations in the recombinant system, there was greater reduction in the second response to cold and menthol in CTNs treated with PMA than in those treated with control solution or 4α-PMA (Figure 4B,C). In CTNs treated with PMA, there was also a shift in the cold threshold to lower temperatures (Figure 4D); note that there was no effect on the threshold in CTNs perfused with control solution or 4α-PM. These results suggest that PKC activation could act as a relevant molecular regulatory mechanism in native membranes, modulating the functional responses of TRPM8-expressing trigeminal cold thermoreceptors.

**Figure 5:**
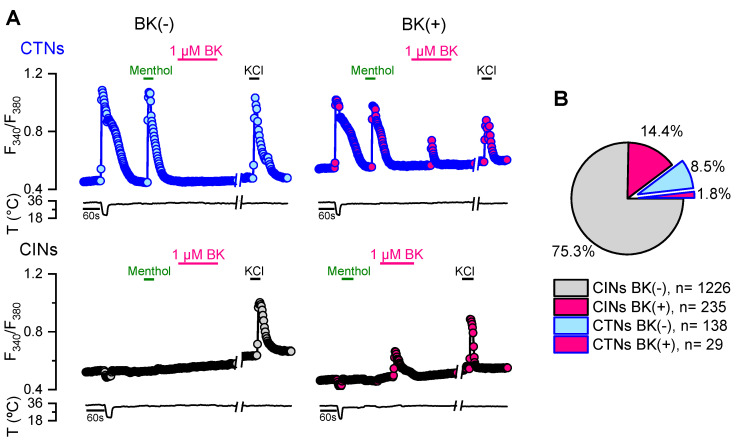
Evaluation of the functional expression of the bradykinin receptors in trigeminal CTNs. (**A**) Protocol in Ca^2+^ imaging used to evaluate the responses of CTNs to cold, menthol (100 µM), bradykinin (1 µM) and KCl (30 mM); CINs are cold-insensitive neurons. The traces correspond to examples of a CTN/BK(−), CTN/BK(+), CIN/BK(−), CIN/BK(+) from TG neurons. (**B**) Pie plot showing the percentage of the different populations of trigeminal primary sensory neurons.

It has been proposed that PKC activation does not participate in the regulation of TRPM8 function mediated by the proinflammatory mediator bradykinin [34,38], although other studies support its contribution [35,37]. The modulation of TRPM8-expressing CTNs by this inflammatory peptide has also been explored in more detail in DRG than in TG neurons. To quantify the CTN population that would be subjected to the modulation of this proinflammatory mediator, we evaluated the functional response to cold and bradykinin from cultured adult TG neurons obtained from intact mice (Figure 5). In agreement with previous studies [48,57], neurons that respond to cooling ramps and 100 µM menthol with [Ca^2+^]_i_ increases represent about 10% (167/1628) of the entire population of trigeminal primary sensory neurons (Figure 5A,B). The percentage of bradykinin-sensitive neurons (i.e., showing an increase in [Ca^2+^]_i_ during bradykinin application due to Ca^2+^-release from InsP_3_-sensitive intracellular deposits) was ~16% (264/1628). Only a small fraction of CTNs exhibited a significant rise in [Ca^2+^]_i_ in response to this proinflammatory mediator (29/167) (Figure 5B), suggesting that the functional expression of bradykinin receptors is restricted to less than 20% of the trigeminal CTNs in these conditions.

### 2.4. PKC Activation Reduces the TRPM8-Dependent Ongoing Activity and Cold-Evoked Responses of Corneal CTNs

**Figure 6:**
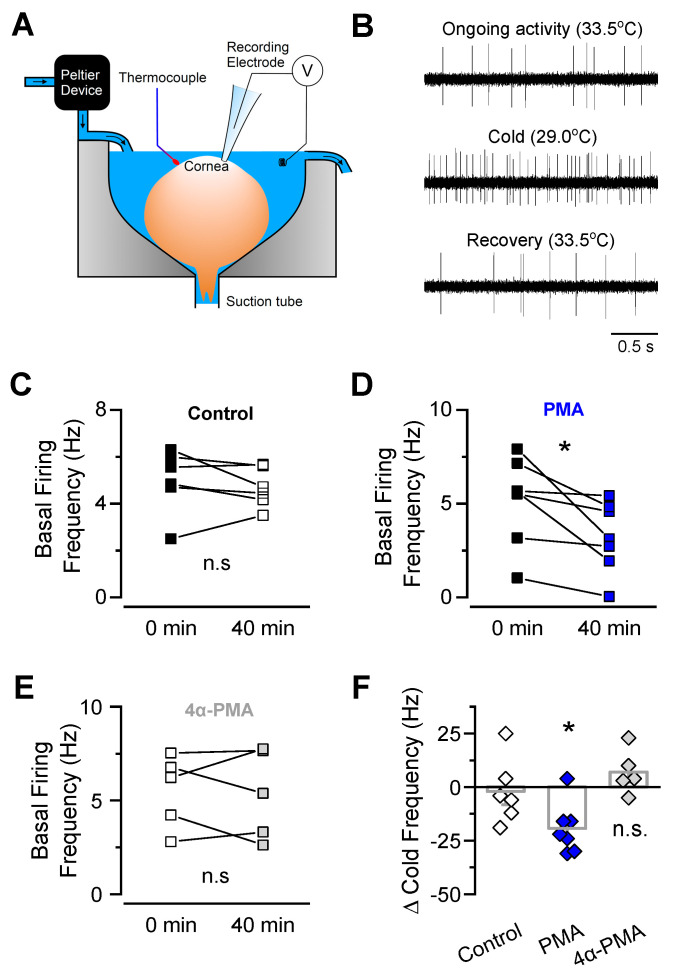
PMA incubation of corneal TRPM8(+) nerve endings reduced their ongoing activity and cold-evoked responses. (**A**) Experimental arrangement of the ex vivo preparation used to record nerve terminal impulse (NTI) activity from corneal cold-sensitive nerve endings in isolated mouse eyes (modified from [5]). (**B**) Examples of 2.5 s of NTI activity recording from a TRPM8(+) cold-sensitive nerve ending in basal conditions, in response to cold, and after rewarming. (**C**–**E**) Ongoing NTI activity before and after application of control solution (**C**), 2 μM PMA (**D**) or 2 µM 4α-PMA (**E**) in cold-sensitive nerve endings. Statistical significance was assessed by paired *t* test; * *p* < 0.05; n.s., not significant. (**F**) Frequency change of NTI activity evoked by consecutive cold pulses from 34 to 20 °C, at time 0 and 40 min, after application of control solution, PMA or 4α-PMA. Data is expressed as the difference between the peak of cold-evoked response at 40 min minus the peak of the response obtained during the first cold pulse in each single nerve ending. Statistical significance was assessed by an ANOVA test in combination with a Dunnett’s post hoc test; * *p* < 0.05; control, *n* = 6; PMA, *n* = 7; 4α-PMA, *n* = 5; n.s. not significant.

Basal tearing production and cold detection are critically dependent on TRPM8 activity in corneal CTNs [5]. To explore the relevance of the PKC-dependent reduction of TRPM8 responses in the mouse cornea, we recorded the electrical activity of corneal cold thermoreceptors ex vivo (Figure 6). In this preparation, nerve endings of cold-sensitive neurons are separated from their somas located in the trigeminal ganglia and preserve their electrophysiological properties (Figure 6A,B). We recorded the ongoing nerve terminal impulses (NTI) activity of single cold thermoreceptors in basal conditions and during cooling ramps, and we determined thermal thresholds to evoke NTI frequency changes (cold threshold) and maximal increases in action potential firing during a temperature drop (maximal cold-evoked response, in spikes by second). After a control period, the cornea was exposed for 40 min to 2 µM PMA or 2 µM 4α-PMA, to allow these drugs to reach the external segment of the corneal nerve fibers. In these conditions, the cooling threshold of corneal CTNs was not changed (control: 32.3 ± 0.3 °C, t = 0, vs. 32.6 ± 0.3 °C, t = 40 min, *n* = 6; PMA: 31.5 ± 0.5 °C, t = 0, vs. 31.9 ± 0.5 °C, t = 40 min, *n* = 7; 4α-PMA: 31.6 ± 0.4 °C, t = 0, vs. 32.6 ± 0.1 °C, t = 40 min, *n* = 5; n.s. *p* > 0.05, paired *t*-tests). Importantly, PMA, but not 4α-PMA, significantly reduced both the ongoing firing activity (Figure 6C–E) and the maximum cold-evoked responses (Figure 6F), suggesting that PKC activation downregulates the basal and cold-evoked electrical activity of corneal CTN nerve endings via modulation of TRPM8 channels in these peripheral sensors.

### 2.5. Mathematical Model of NTI Activity Predicts that the PKC-Mediated Effect on Corneal Nerve Firing Can Be Explained by TRPM8 Downregulation

**Figure 7:**
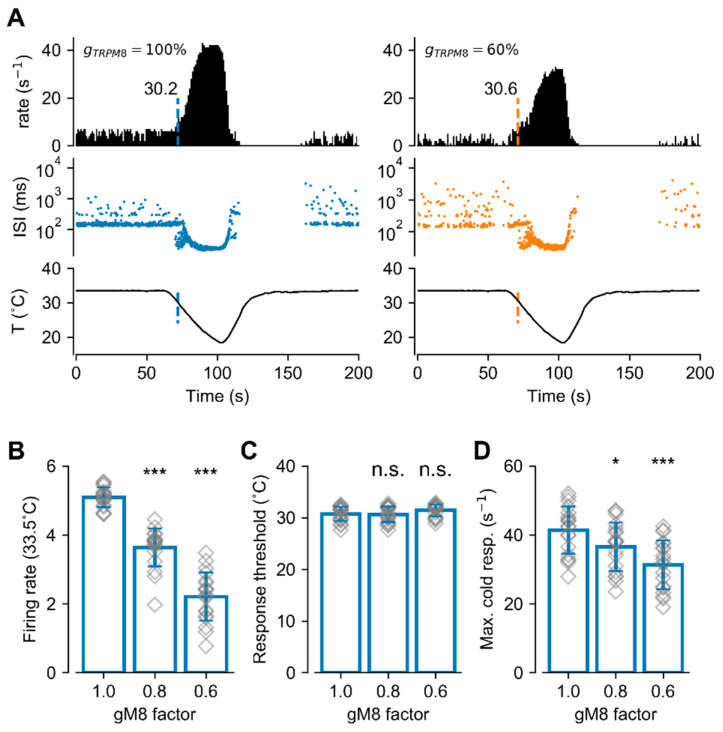
Mathematical model of cold-sensitive nerve endings with different I_TRPM8_ densities. (**A**) Simulated activity of the model exposed to the temperature trace depicted at the bottom, with normal (100%, left) and reduced (60%, right) TRPM8 conductance density. The top row shows the firing rate (spikes/s) and the middle row shows the inter-spikes intervals (ISI; log scale). The vertical colored line shows the moment and the temperature at which the firing rate exceeds the mean plus 3 times the standard deviation of the basal activity (thermal threshold). (**B**) Firing rate at 33.5 °C for 19 sets of parameters (different corneal neurons) (see Table 1), with different densities of TRPM8 conductance. For each model, the normal density of TRPM8 maximal conductance (*gM8* factor = 1.0) is the density that gives a firing rate around 5 spikes/s. (**C**) Temperature threshold for cold-evoked activity increases in the same simulations shown in (**B**). (**D**) Maximum firing rate during the cold pulse, for the simulations shown in (**B**,**C**). In (**B**–**D**), bars represent the mean (±S.D.), and the symbols show the individual data. Each data point is the average of 8 independent simulations with the same set of parameters. * *p* < 0.05, *** *p* < 0.001 and n.s. *p* > 0.05, when compared to the *gM8* factor = 1 condition (Unpaired Student’s *t*-test).

We used our conductance-based mathematical model of corneal CTNs that includes the TRPM8 channel [48,58,59], to emulate the PKC-dependent TRPM8 functional variation and further explore its effect on basal firing and cold-evoked responses. Figure 7A shows the result of simulating the response of our model to a cold pulse, with a normal (left, 100%) or decreased (right, 60%) maximal conductance (g_max_) of the TRPM8 channel, a change similar to the reduction we observed in patch-clamp experiments. The upper panels show the firing rate (spikes/second), the middle panels show the inter-spike intervals (ISI), and the lower panels show the temperature pulse. The cold stimulus causes a dramatic increase in the firing rate, from the basal activity of around 5 spikes/s, and more bursting events (seen in the ISI plot as ISI< 100 ms). When the temperature is brought back to 33.5 °C, there is a transient period of no activity that mimics the experimental recordings [5,48]. When the maximal conductance of TRPM8 (akin to channel density) is diminished to 60%, there is a decrease in the basal firing rate to an average of 2–3 spikes/s. Cold still elicits an increase in the firing rate but the peak of cold-evoked activity is lower than that with the control TRPM8 density. The temperature at which the cold pulse starts to increase firing (i.e., the threshold for cold-evoked activity, indicated by a colored vertical line) did not display significant changes, in agreement with the experimental data from corneal nerve endings.

The simulations described above were repeated with 19 different sets of parameters of the model (Table 1), to mimic different nerve endings and their biological variability. All the parameter sets produce a basal firing rate of around 5 spikes/s (5.0 ± 0.5, similar to the mean ongoing activity observed in the set of corneal nerve endings used in this study), with their control value of TRPM8 maximum conductance (values listed in Table 1). Then, the TRPM8 conductance was decreased to either 80% or 60% of the basal value, and the results are summarized in Figure 7B–D. On average, there is a decrease in basal firing rate (Figure 7B) (at 33.5 °C) and the maximum response to cold (Figure 7D), but no effect on the temperature threshold for the cold-evoked response.

Thus, these in silico results predict that a decrease in functional TRPM8 channels at the plasma membrane of corneal CTNs, as observed during PKC activation, induces an important reduction not only in the ongoing activity of these neurons that could alter basal tear production, but also in their maximal cold-evoked responses, signaling at a less intense temperature drop under an equivalent cold stimulus. Interestingly, reduced TRPM8 conductance does not affect the thermal threshold of these peripheral cold thermoreceptors, in tight correlation with our experimental findings.

## 3. Discussion

This study explores the role of PKC in TRPM8 function. We provide evidence that the PKC activation by phorbol esters reduces the TRPM8 response to cold and menthol, mainly due to a decreased expression of functional channels at the plasma membrane. Our functional evaluation of corneal nerve endings of CTNs shows that their electrical activity can be reduced by pharmacological activation of PKC, suggesting that this form of modulation should not be overlooked in studies assessing not only physiological but also physiopathological scenarios, such as those triggered by a local inflammatory process.

To date, the role of PKC as a modulator of TRPM8 function is controversial, even in cells treated with PDBu or PMA, where only PKC activation should be considered in the equation [34,35,37,45,46,54,60]. In that regard, it is important to highlight that studies reporting a lack of effect of PMA [34,54] were mainly focused on dissecting the role of PKC in a specific downregulation/desensitization mechanism, rather than assessing the ability of this molecule to modulate TRPM8 function per se. Therefore, the time course of treatment with PKC activators or the specific experimental approach used by different groups could contribute to these discrepancies. One example is the temperature conditions used in functional experiments, a key factor for a thermo-TRP channel such as TRPM8. Zhang and colleagues reported that measured Ca^2+^ response ratios to 100 µM menthol before and after two minutes of treatment with 1 µM PMA were similar to those from cells only exposed to control solution at room temperature [34]. In the protocol used here (Figure 1F and Figure 4A), the basal temperature of our experiments was 34 °C. This difference has to be considered, since our results showed a robust reduction with PMA treatment only when responses to one stimulus (cold (from 34 to 20 °C)) or menthol (100 µM menthol at 34 °C) were assessed, but were non-significant when co-stimulation with cold plus menthol (100 µM menthol at 20 °C) was performed (see Figure 1B,D,E). This suggests that the impact of PKC activation on TRPM8 function can be underestimated by Ca^2+^-imaging when a saturating stimulus is applied. Considering that Zhang et al. investigated the effects of menthol at room temperature (a state closer to our cold plus menthol condition, i.e., 100 µM menthol at 20 °C), it is possible that a reduced channel response induced by PMA could have been overlooked. However, that could not be the only explanation, since a similar approach was used in a different report showing TRPM8 modulation induced by phorbol esters [45]. One additional difference is the shorter incubation time compared to the 5 to 10 min that we and others used during these experiments [37,45,46,60]. The conditions and the experimental procedure used by Zhang and coworkers to test PMA-dependent PKC activation were justified on the grounds that they were equivalent to those where the downregulation exerted by bradykinin was assessed [34]. However, these experiments did not rule out that PKC-activation could modulate TRPM8 function in a different set of conditions.

Yudin and coworkers reproduced the inhibitory effect of PMA on menthol-evoked channel responses in the absence of extracellular Ca^2+^ using whole-cell patch-clamp recordings [54]. Nevertheless, they observed no major changes in desensitization levels under normal Ca^2+^ in the extracellular milieu, concluding that PKC does not participate in the TRPM8 Ca^2+^-dependent menthol-induced desensitization mechanism [54]. However, in our Ca^2+^-imaging experiments, both TRPM8-expressing HEK-293 cells and CTNs incubated with PMA exhibited an additional negative modulation above the desensitization observed in cells treated with control solution or 4α-PMA (see Figure 1 and Figure 3). Since Yudin and coworkers performed TRPM8 stimulation using 500 µM menthol at 22–24 °C, one possible explanation is that the larger desensitization elicited in these saturating conditions could mask, at least in part, the contribution of PKC modulation.

It is important to highlight that important information to fully unravel the PKC-dependent pathway involved in the regulation TRPM8 function is still required (reviewed by [61]). Premkumar and coworkers reported that PKC-induced phosphatase activity is necessary to modulate TRPM8 function [37], suggesting that PKC is one of the steps of a more complex regulatory signaling cascade. Therefore, the entire picture of the molecular components that take part in this pathway is still incomplete. A recent study reported that chronic exposure to morphine prevented TRPM8 desensitization via PKCβ in DRG neurons [62], indicating that further studies exploring the contribution of specific PKC isoforms in subpopulations of TRPM8-expressing neurons could also reveal important differences in the effects and the molecular mechanisms involved in PKC-dependent regulation of TRPM8 in primary sensory neurons.

The reduced g_max_ and the number of active TRPM8 channels assessed by noise analysis revealed that PKC activation induces a ~40% drop in functional channels at the plasma membrane. It is known that the TRPM8 channel travels to the plasma membrane of primary sensory neurons in atypical secretory vesicles with lysosomal-associated membrane protein 1 (LAMP1) and RAB7 molecular identity [63]. Recently, we also described a non-conventional trafficking mechanism involved in the recycling of TRPM8 channels to the plasma membrane in sensory fibers, with an important role in peripheral cold sensing [52]. Our present findings suggest that PKC could alter TRPM8 trafficking, as is the case for several ion channels and transporters [64,65,66]. The total amount of TRPM8 channels at the plasma membrane is the result of synthesis, exocytosis, endocytosis, and degradation dynamics. Based on the fact that the PMA-mediated reduction of TRPM8 channels is observed after ten minutes of treatment, it is unlikely that a reduction in TRPM8 synthesis is behind this process. We speculate that the reduction could be the result of alterations in the endocytosis and/or recycling rate of the channel. However, other explanations should be also taken into account, considering the lack of PMA-dependent internalization of TRPM8 that Abe and co-workers described using a biotinylation assay [46]. In this study, we used a functional approach to assess the expression of TRPM8 in the plasma membrane; therefore, it could be also possible that stabilization of non-conducting states of TRPM8 takes place as a result of PKC activation, explaining the drop of functional channels at the cell surface without an actual decrease in the TRPM8 protein levels in this cellular compartment.

The functional roles of corneal CTNs are diverse. In addition to their perceptual role, TRPM8(+) cold thermoreceptors that innervate the cornea act as ocular wetness sensors, detecting evaporation-evoked temperature and osmolality oscillations, which finally set basal tear secretion and blinking rates, thereby preventing desiccation of the eye surface and contributing to the maintenance of corneal homeostasis [5,6,7,9]. These functions of corneal CTNs are strongly dependent on the expression of TRPM8 channels in their peripheral nerve endings, without a significant contribution of other cold-sensitive channels such as TRPA1 [5,6,9]. Corneal CTNs often fire action potentials in a regular pattern at the normal temperature of the corneal surface (~33 °C), which markedly increases with small temperature drops (<1 °C) [5,67,68,69], tonically modulating basal tearing and spontaneous blinking [5,6,70,71]. Thus, subtle disturbances in the expression, trafficking, local modulation and/or temperature-dependent activation of TRPM8 in these neurons could lead to membrane excitability changes with highly relevant physiological consequences. Our results using PMA showed an important drop in the spontaneous firing of CTNs at basal temperature. In the physiological context of corneal humidity control, this change could be enough to significantly reduce the basal tearing rate, modifying the tear film properties and potentially affecting the integrity of the corneal surface [9]. In these neurons, downregulation of TRPM8 markedly reduced the maximal response to cold stimuli but had minor effects on the thermal threshold. Remarkably, all the functional modifications on the electrical activity of nerve endings were well reproduced by our mathematical model of corneal CTNs, where TRPM8 was the only molecular target affected by PKC activation during the simulations mimicking the effect of this kinase, i.e., a 40% reduction in the g_max_ of TRPM8. Especially relevant is the prediction that the thermal threshold of these individual fibers is poorly sensitive to variations of TRPM8 channel expression compared to the effect on the ongoing activity and maximal cold-evoked responses (see Figure 7). Although we cannot discard the effect of PKC activation on other channels involved in the modulation of impulse firing in corneal nerve endings, our mathematical modeling results suggest that a reduction in TRPM8 channels alone can explain the firing phenotype we observed in treated terminals, reinforcing the relevance of TRPM8 activity in the excitability changes evoked by PKC activation.

Sensory information provided by cold-sensitive nerve terminals expressing TRPM8 is involved not only in the conscious sensation of cold, but also in the perception of warmth and pain [10,11,12,72]. Therefore, altered TRPM8 function as the result of peripheral nerve damage and/or inflammation processes could lead to sensory alterations such as cold allodynia and hyperalgesia [20,21,22,23]. In this scenario, numerous signaling pathways activated by tissue damage could converge toward the activation of PKCs causing pain (reviewed by [51]), where one of the main mechanisms involved is the PKC-dependent sensitization of TRPV1 channels [73,74,75]. However, considering that TRPM8 activation by cooling or menthol application induces an analgesic effect [10,76], it has also been suggested that downregulation of this channel could intensify the pain condition, resulting in a sensitized phenotype [34,37].

As was discussed previously, several reports established that the release of inflammatory mediators causes a decrease in cold- and menthol-evoked responses in DRG neurons treated through the activation of their cognate G-protein-coupled receptors [33,34,35,37,38]. The contribution of PKC activation towards bradykinin’s negative modulation of TRPM8 function has been questioned [34,38]. However, considering that inflammation could lead to PKC activation by different signaling pathways (reviewed by [51]), and functional expression of the bradykinin receptor occurs only in a subpopulation of CTNs in the trigeminal ganglia (see Figure 5B and [33]), a contribution of a PKC-dependent modulation of cold-evoked responses in CTNs should not be fully ruled out in inflammatory conditions. This could be relevant in peripheral axons and nerve terminals of corneal sensory neurons, that are often exposed to inflammation induced by the use of contact lenses, surgical procedures or dry eye disease, to name a few [47]. Thus, understanding the complex scenario derived from local inflammation could be essential to design effective therapeutic strategies that take into account the role of TRPM8 channels.

In summary, our results suggest that the activation of PKC by PMA is sufficient to downregulate TRPM8 function. We also demonstrate that this regulation takes place in CTNs, modulating their response to cold, and suggest that this particular form of dynamic regulation should be considered to fully understand some pathological conditions based on functional alterations of TRPM8 channel function.

## 4. Materials and Methods

### 4.1. Animals

This study was performed using male and female young adult (P21–P40) C57BL/6 mice. Animals were housed at a maximum of four per cage in a 12 h light/dark cycle, with food and water ad libitum, and euthanized with CO_2_. All experiments were conducted according to the bioethical guidelines of the Comisión Nacional de Investigación Científica y Tecnológica de Chile (CONICYT) and have been approved at 26 April 2018 by the Bioethical Committee of the University of Santiago de Chile (reference number 289/2018).

### 4.2. Cell Culture

Mice were sacrificed by CO_2_ inhalation. After decapitation, trigeminal ganglia (TG) were removed and incubated in an enzymatic mixture in INC-mix solution (in mM: 155 NaCl, 1.5 K_2_HPO_4_, 10 HEPES, 5 Glucose, pH: 7.4) containing collagenase type XI (650 UI/mL; C7657, Sigma-Aldrich, St. Louis, MO, USA) and dispase (5 UI/mL; 17105-041 GIBCO-Thermo Fisher Scientific, Waltham, MA, USA), for 40 min at 37 °C in 5% CO_2_. The ganglia were then mechanically dissociated with polished Pasteur pipettes and neurons were plated on poly-l-lysine-coated 6 mm #0 glass coverslips (Menzel-Gläser, Braunschweig, Germany). Cultured trigeminal neurons were maintained in MEM media (Earle’s salts, 11095080, GIBCO-Thermo Fisher Scientific, Waltham, MA, USA) supplemented with MEM-vit (11120052, GIBCO-Thermo Fisher Scientific, Waltham, MA, USA), 10% FBS (SH30910.03, Hyclone, General Electric Healthcare Life Science, Logan, UT, USA), 200 µg/mL streptomycin, 125 µg/mL penicillin (15140-122, GIBCO-Thermo Fisher Scientific, Waltham, MA, USA), and used within 6 to 12 h for [Ca^2+^]_i_ imaging and patch-clamp recordings.

### 4.3. Recombinant Expression of TRPM8

A HEK-293 cell line stably expressing mouse TRPM8-myc channels (HEK-293-mTRPM8-myc cells) [52] was used in this study. This cell line was cultured in DMEM containing 10% fetal bovine serum and 600 µg/mL geneticin. Twenty-four hours before Ca^2+^ imaging or patch-clamp experiments, cells were trypsinized and seeded on poly-l-lysine-coated 6 mm #0 glass coverslips (Menzel-Gläser, Braunschweig, Germany).

### 4.4. Ca^2+^-Imaging

Ca^2+^-imaging experiments were conducted with fluorescent indicator Fura-2 AM (F1221, Invitrogen-Thermo Fisher Scientific, Waltham, MA, USA). Before each experiment, the cells were incubated with 5 μM Fura-2AM for 50 min at 37 °C in standard extracellular solution supplemented with 0.02% Pluronic acid (P6867, Invitrogen-Thermo Fisher Scientific, Waltham, MA, USA). The standard extracellular solution, referred to as control solution, contained (mM): NaCl 140, KCl 3, CaCl_2_ 2.4, MgCl_2_ 1.3, HEPES 10 and glucose 10, and was adjusted to pH 7.4 with NaOH. Fluorescence measurements were obtained using an inverted Nikon Ti microscope equipped with a Super Plan Fluor ELWD 20XC objective N.A. 0.45 (Nikon Instruments Inc., Melville, NY, USA) and a 12-bit cooled ORCA C8484-03G02 CCD camera (Hamamatsu, Hamamatsu City, Japan). Fura-2 was excited at 340 and 380 nm with a Polychrome V monochromator (Till Photonics, Munich, Germany), with exposure times no longer than 40 ms; the emitted fluorescence was filtered with a 510 nm long-pass filter. Calibrated ratios (at 0.5 Hz) were displayed online with HCImage v2 software (Hamamatsu, Hamamatsu City, Japan). Bath temperature (see details below) was sampled simultaneously using a BAT-12 microprobe thermometer (Physitemp Instruments, Clifton, NJ, USA) supplemented with an IT-18 T-thermocouple, using Clampex 10 software (Molecular Devices, San Jose, CA, USA). The signal was digitized with an Axon Digidata 1440A AD converter (Molecular Devices, San Jose, CA, USA).

Threshold temperature values for the rise in [Ca^2+^]_i_ were estimated as in [77]. For this, the temperature was linearly interpolated at the midpoint between the baseline and the first point at which [Ca^2+^]_i_ elevation deviates by at least four times the standard deviation of the baseline.

### 4.5. Patch-Clamp Recordings

Whole-cell voltage-clamp recordings in transfected HEK-293 cells were performed simultaneously with temperature recordings. Standard patch pipettes (3–5 MΩ) were made of borosilicate glass capillaries (Harvard Apparatus Ltd., Cambridge, UK) and contained (in mM): 130 CsCl, 1 EGTA, 10 HEPES, 4 ATP-Mg, and 0.4 GTP-Na, pH adjusted to 7.4 with CsOH (280 mOsm/kg). The bath solution was the same solution used in the Ca^2+^ imaging experiments. Current signals were recorded with an Axopatch 200B patch-clamp amplifier (Molecular Devices, San Jose, CA, USA). Stimulus delivery and data acquisition were performed using pClamp10 software (Molecular Devices, San Jose, CA, USA). To estimate the shifts in the voltage dependence of TRPM8 activation, current-voltage (I–V) relationships obtained from repetitive (0.2 Hz) voltage ramps (−100 to +180 mV, with a slope of 200 mV/s) were fitted with a function that combines a linear conductance multiplied by a Boltzmann activation term [78]:

*I* = *g* × (*V* − *E_rev_*)/(1 + *exp*[(*V*_1/2_ − *V*)/*s*]) (1)

where *g* is the whole-cell conductance, *E_rev_* is the reversal potential of the current, *V*_1/2_ is the potential for half-maximal activation and s is the slope factor. Linear conductance is assumed based on the observation by [16] that open TRPM8 channels exhibit an ohmic I–V dependence.

### 4.6. Temperature Stimulation

Coverslips with cultured cells were placed in a microchamber and continuously perfused with solutions warmed to ~34 °C. The temperature was adjusted with a water-cooled Peltier device that was computer-controlled and placed at the inlet of the recording chamber, and controlled by a feedback device. Cold sensitivity was evaluated with temperature drops from ~34 to 20 °C.

### 4.7. Variance Analysis

To estimate the number of active TRPM8 channels in the plasma membrane, we used non-stationary noise analysis [79]. A total of 100 current records in whole-cell configuration were collected for each cell, during activation of the channels, by 150 ms depolarizing voltage steps from 0 to +180 mV, at 19 °C. Ensemble averaged current (<*I*>) and its variance (*σ*^2^) on each isochrone were calculated. The variance as a function of <*I*> was fitted using the equation:

*σ*^2^ = i*<*I*> − (*<I*>^2^/*N*) (2)

where *i* is the single-channel unitary current and *N* is the number of channels in the plasma membrane. The maximum open probability (*Po*_max_) was estimated using the relation *Po*_max_ = *I*_max_/i*N, where *I*_max_ is the mean maximal current in each experiment. Data for variance analysis were acquired at 20 KHz and filtered at 5 KHz.

### 4.8. Extracellular Recordings of Corneal CTNs

Extracellular recording of nerve terminal impulse (NTI) activity in vitro was performed as in [5]. In brief, the eyes were carefully removed from the sacrificed animals and placed in a 25 mL glass containing oxygenated extracellular solution. Excised eyes were then placed in the recording chamber, and the optic nerve and associated tissues were drawn into a suction tube at the bottom of the chamber, and continuously perfused (1 mL/min) with a physiological saline solution of the following composition (in mM): 128 NaCl, 5 KCl, 1 NaH_2_PO_4_, 26 NaHCO_3_, 2.4 CaCl_2_, 1.3 MgCl_2_ and 10 glucose, pH 7.4, gassed with carbogen (95% O_2_, 5% CO_2_). The basal temperature of the bath solution was kept at 33–34 °C, and was modified using a CS-1 Temperature Controller (Cool Solutions Research Devices, Carrigaline, Ireland) controlled by a computer, and the outlet was located close to the surface of the eye. A glass pipette (tip diameter: ~50 to 100 µm) for recording extracellular NTI activity, filled with physiological saline, was positioned onto the corneal epithelium surface and slight suction was applied. Signals were amplified with an 1800 AC amplifier (A-M Systems, Carlsborg, WA, USA), and data were acquired and analyzed using an Axon 1332A Digidata AD converter (Molecular Devices, San Jose, CA, USA) coupled to a computer running pClamp 9 software (Molecular Devices, San Jose, CA, USA). Further analysis was performed using Spike2 8.0 software (Cambridge Electronic Design, Milton, Cambridge, UK). Only nerve impulses that were readily distinguished from noise (~10 µV peak-to-peak when low-pass filtered at 5 kHz) and with similar shape and amplitude were studied. 

Cold thermoreceptor nerve endings were identified by their typical spontaneous, often regular, low-frequency impulse activity at 34 °C, which increased during temperature drops and were transiently silenced by re-warming. Ongoing NTI activity (spontaneous firing activity) at 34 °C was recorded for at least 3 min before cooling. Basal mean ongoing activity (in impulses per s) was calculated during the 30 s preceding the onset of a ~30 s ramp-like temperature drop to 20 °C at a rate of ~0.7 °C/s. This protocol was repeated during exposure to PMA. The temperature threshold for the cooling stimulus corresponds to the temperature at which NTI frequency increased to a value that was the mean NTI frequency, measured during the 10 s period that preceded the onset of the cooling pulse, plus three times its standard deviation.

### 4.9. Mathematical Model

To corroborate the correlation between firing frequency and TRPM8 functional expression level, we used our CTNs model including TRPM8, first described in [58,59]. The equation for the membrane potential is as follows:

(3) $$C_{m}\frac{dV}{dt}=-I_{sd}-I_{sr}-I_{d}-I_{r}-I_{M8}-I_{l}+I_{wn}$$

where *C_m_* is the membrane capacitance, *I_sd_* and *I_sr_* are the slow depolarizing and repolarizing currents, respectively, that create the intrinsic oscillation of membrane potential. *I_d_* and *I_r_* are Hodgkin and Huxley-type depolarizing and repolarizing currents for action potential firing, *I_M_*_8_ is the TRPM8-dependent cold-activated current, *I_l_* is an ohmic leakage current and *I_wn_* is a noise term. Ionic currents are given by:

(4) $$I_{i}=g_{i}a_{i}\left(V-E_{i}\right)\;\;\;\;\;\;\;\left(i=sd,sr,d,r,M8,l\right)$$

where *g_i_* represents the maximum conductance density of the current *i*, given by the level of channel expression. *E_i_* is the reversal potential of the current and *a_i_* is the activation variable or open channel probability, usually voltage-dependent, except for $a_{l}\equiv 1$ (for other equations and details see [58,59]). The I_KD_ current [77,80] is contained in the rest of the parameters considered in the model, and therefore it is invariant in all simulations [48].

As in our previous work [48,59], we employed different sets of parameters that give different types of dynamic responses. The sets used in this study are listed in Table 1, and the common parameters are:

$$\begin{array}{l} C_{m}=1\;\left(\frac{\mu F}{{\mathrm{cm}}^{2}}\right);\;E_{l}=-70,\;E_{d}=E_{sd}=50,\;E_{r}=E_{sr}=-90,\;E_{M8}=0\;\left(\mathrm{mV}\right);\\ \tau_{sd}=10,\;\tau_{sr}=24,\;\tau_{r}=1.5\;\left(\mathrm{ms}\right);\;s_{sd}=0.1,\;s_{d}=s_{r}=0.25\;\left({\mathrm{mV}}^{-1}\right);\\ V_{sd}^{h}=-40,\;V_{d}^{h}=V_{r}^{h}=-25\;\left(\mathrm{mV}\right);\;\eta =0.012\;\left({\mathrm{cm}}^{2}/\mu A\right);\;\kappa =0.17;\;z_{M8}=0.65;\\ \Delta E=9000\;\left(J\right);\;K_{Ca,M8}=0.5\;\left(\mu M\right);\;d=1\;(\mu m);\;D=0.5\;\left(\mu A/{\mathrm{cm}}^{2}\right);\;\tau_{wn}=1\;(\mathrm{ms}). \end{array}$$

The model was implemented in the Neuron simulation environment (RRID: SCR_005393) controlled with Python scripts (RRID: SCR_008394) [81,82]. Analysis of the simulations was performed in Python with the libraries Numpy (RRID: SCR_008633), Scipy (RRID: SCR_008058), and Matplotlib (RRID: SCR_008624).

### 4.10. Reagents and Drugs

L-menthol (Menthol), PMA and 4α-PMA were purchased from Sigma-Aldrich (St. Louis, MO, USA).

### 4.11. Data Analysis

Data are reported as mean ± S.E.M. (standard error of the mean) or mean ± S.D. (standard deviation) from n cells studied. When comparing two mean values, statistical significance (*p* < 0.05) was assessed using Student’s paired or unpaired, two-tailed *t* test. For multiple comparisons of means, one-way ANOVA was performed in combination with a Bonferroni’s or Dunnett’s post hoc test. Data analyses were performed using PRISM™ 5 (GraphPad Software, Inc., San Diego, CA, USA).

## Figures and Tables

**Table 1:** Parameter sets used in the model.

	$g_{M8}$	$g_{l}$	$g_{sd}$	$g_{sr}$	$g_{d}$	$g_{r}$	$p_{Ca}$	$\tau_{Ca}$	$\tau_{\delta V}$	$\delta V_{min}$	$\delta V_{max}$
Set #	$mS/cm^{2}$						$\times 10^{4}$	ms		mV	
1	2.65	0.27	0.29	0.20	3.7	5.0	1.8	39,000	2167	−160	215
2	1.61	0.24	0.28	0.22	3.5	4.9	2.5	45,833	2083	−220	170
3	1.03	0.21	0.35	0.31	3.0	4.4	1.3	40,000	5167	−230	250
4	0.46	0.17	0.21	0.28	4.0	4.9	4.7	23,333	13,667	−250	110
5	0.58	0.16	0.20	0.28	3.9	4.7	5.2	23,333	16,000	−225	150
6	4.02	0.24	0.30	0.25	4.0	5.0	3.5	33,333	2167	−230	185
7	4.95	0.22	0.25	0.21	3.9	5.0	3.2	66,667	5833	−150	170
8	1.50	0.21	0.28	0.26	3.8	4.7	3.6	43,333	6667	−250	150
9	4.03	0.27	0.32	0.20	2.8	4.9	3.4	39,167	8333	−190	235
10	3.80	0.26	0.33	0.21	3.0	4.7	3.3	65,000	15,333	−220	250
11	3.69	0.18	0.21	0.23	2.5	3.4	4.6	40,833	11,667	−230	240
12	2.30	0.19	0.21	0.22	2.7	3.0	4.7	31,667	25,000	−230	250
13	3.11	0.20	0.21	0.20	2.4	2.3	5.5	40,000	13,833	−250	230
14	1.50	0.28	0.34	0.20	3.3	4.7	1.4	63,333	6833	−140	240
15	2.30	0.29	0.34	0.20	3.0	4.2	4.8	35,833	2333	−210	170
16	1.61	0.28	0.34	0.20	3.1	5.0	3.8	30,000	9000	−150	190
17	2.99	0.27	0.33	0.21	2.8	3.7	5.4	26,667	15,167	−140	170
18	1.84	0.23	0.25	0.20	4.0	5.0	5.8	31,667	10,417	−220	170
19	3.45	0.23	0.25	0.20	4.0	5.0	5.8	31,667	10,333	−250	250

*g*_*M*8_, *g_l_*, *g_sd_*, *g_sr_*, *g_d_* and *g_r_* are the maximum conductance densities of the respective ion currents (representative of the ion channel expression level). *p_Ca_* is a parameter that controls how much the TRPM8 current contributes to an increase in [Ca^2+^]_i_ and channel desensitization. *τ_Ca_* and *τ_δV_* are the time constants for Ca^2+^ removal and for the desensitization process, respectively. *δV_min_* and *δV_max_* are the minimum and maximum values for TRPM8 V_1/2_ displacement due to Ca^2+^-dependent desensitization (see [58,59]).

## Abbreviations

CTN	Cold Thermoreceptor Neuron
PKC	Protein Kinase C
PMA	Phorbol 12-Myristate 13-Acetate
TG	Trigeminal ganglia
DRG	Dorsal Root Ganglia
TRPM8	Transient Receptor Potential Melastatin 8
TRPV1	Transient Receptor Potential Vanilloid 1
BR2	Bradykinin Receptor 2
PIP_2_	Phosphatidylinositol 4,5-bisphosphate
InsP3	Inositol 1,4,5-trisphosphate
PDBu	Phorbol 12,13-Dibutyrate
NTI	Nerve Terminal Impulse
